# Dr. Mayo on Popular Superstitions

**Published:** 1849-10-01

**Authors:** 


Art. II.—*Letters on the Truths contained in Popular Superstitions.* By Herbert Mayo, M.D. 1849. Sauerlænder, Frankfort; Blackwood, Edinburgh.

The present era is distinguished for the development both of physical and psychical wonders. In mechanical and other arts, what miracles have been wrought by the ingenuity and scientific experiments of the engineer and the practical chemist; the tunnelling of iron-stone rocks; the draining of primæval bogs and mosses; the suspension, as if by magic, of gigantic tubes over the wide waters; the lightning celerity of the electric telegraph, all demonstrate the wondrous workings of the brain of man, and the perfection of his hand.

The elucidation of the eccentric phenomena of mind seems almost to have kept pace with the inventions of science, and has rendered clear and evident many of those mysterious influences resulting from the combination of spirit and matter which were, in darker ages, referred to nought less than the special agency of a deity or a demon.

It may be with the imaginative classes of mankind a sort of profanation to invade those fanciful regions, which have become almost sacred from their very antiquity, to throw a cloud of dull fact over the bright regions of fairyland; but truth is, or should be, the goddess of our idolatry, and it is our duty as philosophers to remove the veil of obscurity from the portal of her temple, and thus weed the human heart of its superstitious errors. "To call a prejudice time-hallowed," writes Lord Bacon, "is to open a way for it into hearts it had never before penetrated. Thus it is with human belief."

We have in this notice presumed to place mechanics and mind in the same category, for their mutual influence is, in more senses than one, in very intimate association.

We do not, of course, for the present, allude to the preconception of a theory or a principle in the mind, ere a *work* can be effected by the hand; this is a truth too plain to require even a momentary argument.

The imaginative faculty of mind, however, has, from time to time, excited more influence over mechanical invention than we may at first believe. Thus in the infancy of science every mind which could not compass or fathom the principles of an eccentric feat or a gigantic invention, at once charged the inventor with the possession of supernatural power, and of having, for evil purposes, entered into league with a demon. The vulgar and illiterate, nay, many a cultivated mind, were prone to blink an extraordinary work, unless they were satisfied that extraordinary—that is, supernatural—powers were brought into play to effect it. To this feeling Galileo owed his persecution; thus, too, when Roger Bacon had invented a magic lanthern, no one believed it, until they were assured that the devil appeared to, and assisted him, (for they gave implicit faith to the sable impersonation,) and then their scepticism vanished.

This blind credence in the mechanical or personal agency of the devil upon earth was one fertile spring of those popular superstitions which have for ages deluged the globe. They have blinded the eye of the mind to the light of truth, and they have done what is far more to be deplored, they have stained the human heart with black infidelity, and, under the guise of priestly sanctity, with cruelties the most wanton and devilish. While by some, the pure light of revelation has been scorned as an ignis fatuus to the weak proselyte of divine truth; by others, the believer has been agonized with tortures indescribable, in obedience to the fiat of a wanton nun or a bigoted friar.

For ages these superstitions were hoodwinked; learning and science, if such it can be termed, were in the hands of the magi or the priests, who, at one time, even vaunted a delegated power and authority from above, by which they worked their miracles.

As time has progressed, the light of science has been more spread abroad, and has entered deeply into men's minds; the dark veil of superstition has been torn away, and the enlightened intellect can now analyze the principles of nature, and without fear elucidate and explain her wondrous and beautiful laws. Hence is Herbert Mayo fully justified, among others, in writing on the *truths* of popular superstitions.

These letters were written as "a resource in the solitary evenings of commencing winter;" but the thrilling delights of juvenile imagination did not, to his regret, come over the spirit of the author's dream. He was out of the leading strings of fancy, and had become a man of fact; and therefore, instead of a running fire about the wild legends of the Hartz or the Katzenellenbogen or the Lurleiberg, the psychologist has boldly shorn them of their romantic interest, and they dwindle, as Gafferel has it, into "very truths, naturally falling under the compass of his matter."

With all these elucidations, however, the world is still composed, with regard to psychical phenomena, of three classes; the sceptics or revilers at one extreme, the proselytes or believers at the other, and between them the reflectors; who, though still in various degrees doubting, yet have their mind's eye open to conviction, being assured that, although some professors deceive themselves, and some deceive others, there are yet "more things in heaven and earth than are dreamt of in our philosophy."

The subjects Mr. Mayo has chosen for his analyses are these—the divining rod, vampyrism, ghosts, real and unreal, trance, somnambulism, catalepsy, religious delusions, mesmerism: a very limited catalogue, it must be confessed, on which to build an analysis of superstition, although, in the form of letters in Blackwood, they passed off as light and interesting articles.

The style in which the work is written savours much of the mysticism of the German school, and it is printed in an outlandish type. This, added to the *un-English* spelling of such words as *phraze, exercize,* &c., causes us to feel that the literary qualities of Mr. Mayo are not improved by his residence at Boppard on the Rhine.

The subjects have been already well sifted in the Natural Magic of Brewster, the Sciences Occultes of Salvertes, the Essays of Pettigrew and of Newnham, the Popular Delusions of Mackay, and in Dendy's "Philosophy of Mystery," &c., and most of the incidents have already been recorded in one or other of those works; yet as every year now bringeth forth something novel—as Mr. Mayo dilates more fully than many others on the wondrous O. D. force, that great natural spell of the world, that so completely unmasks the magus and the wizard—as the author has been an authority in physiology—and as, above all, the book is a *little* one, we have now chosen it as the subject of our comments.

Among the popular superstitions of the mining districts there is one which confers the power of discovering the hidden treasures of the mines—a faculty which would have once secured to its possessor the title of magus or impostor; and he would have been worshipped or murdered—have had his apotheosis or his bonfire, according as his works called up proselytes or scorners. But the magic is not really of the mind, it seems, not even of the sensations; it is purely mechanical, the mind being merely concerned in interpreting or calculating the sources of action—the movements of the *divining rod,* to which eccentric phenomena Mr. Mayo gives a qualified credence.

"There exists strong evidence to show that the divining rod really does what is pretended of it." And this is the account of the talisman:

"In Cornwall, they hold that about one in forty possesses this faculty. They cut a hazel twig just below where it forks. Having stripped the leaves off, they cut each branch to something more than a foot in length, leaving the stump three inches long. This implement is the divining rod. The hazel is selected for the purpose because it branches more symmetrically than its neighbours. The hazel fork is to be held by the branches, one in either hand, the stump or point projecting straight forwards. The arms of the experimenter hang by his sides; but the elbows, being bent at a right angle, the forearms are advanced; the hands are held seven or eight inches apart, the knuckles down, and the thumbs outwards. The ends of the branches of the divining fork appear between the roots of the thumbs and forefingers. The operator thus armed walks over the ground he intends exploring, in the full expectation that, if he possesses the mystic gift, as soon as he passes over a vein of metal or under-ground spring, the hazel fork will begin to move spontaneously in his hands, rising or falling as the case may be."

Mr. Mayo's conversion was, however, a roundabout sort of an influence. A Scotch gentleman, Mr. G. Fairholme, told Mr. Herbert Mayo that Mrs. H——, of Southampton, told him that Mrs. Colonel Beaumont, of Cheltenham, told her that she could show her the effect of the rod, which she did in a shrubbery; but no mention of the discovery of a mine. Even the experiments of the Count de Tristan, who wrote a book on the subject, do not come up to the mark, nor exactly elucidate the manœuvres of Dousterswivel in the chapel of St. Ruth, as the rod will, in certain degrees, move over a

*non-exciting* surface, being affirmed to be dependent on electricity, that prolific explanation (?) of many influences, as mesmerism, cholera, influenza, and the like. The sum of Mr. Mayo's conclusions is, that we must be *predisposed* ere we can influence the divining rod; that we are not conscious of our influence; that it is only shown in certain localities; that the rod is, in fact, only a conductor between the body and the earth.

As an analogous power to this, the author refers to the magnetic experiments of Reichenbach, by which a new force was imparted which is termed the O. D. This O. D. (D.) seems to be but another reading of the magnetism of Puysegur many years ago; yet the phenomena being well authenticated, we will quote some passages of interest.

"A horse-shoe magnet having been adjusted on a table, with the poles directed upwards, the sensitive subject saw, at the distance of ten feet, the appearance of flames issuing from it. The armature of the magnet, a bar of soft iron, was then applied. Upon this the flames disappeared. They reappeared, she said, as often as the armature was removed from the magnet."

It seems that the O. D. is a *polar* force; its effects being, according to Von Reichenbach, O. D. *negative* and O. D. *positive.*

"One of the most sensitive of his subjects held, at his desire, a piece of copper-wire, by the middle with the right hand, by one end with the left. Then Von R. touched the free end of the wire with one pole of a large crystal, in order to charge it with O.D. The patient immediately felt a sensation in the right hand, which disappeared as quickly to be felt by the left hand instead, at the further end of the piece of wire."

"The right hand displays the characters of negative O. D., the left those of positive O.D. The more sensitive subjects recognised in the dark the appearance of dim flames proceeding from the tips of his fingers, and all felt the corresponding sensations of draughts of cool or warm air."

"Von Reichenbach substituted himself for the magnet. When he took Miss Maix's hands in his normally—that is to say, her left in his right, her right in his left, she felt a circulation moving up the right arm through the chest, down the left arm, attended with a sense of giddiness."

Now these incidents, taken with reservation, are interesting, yet we are still sceptical enough to smile at the assurance of "flame proceeding from the tips of Von Reichenbach's fingers," as when we first heard from the lips of Dupotet the same assertion in the wards of the Middlesex Hospital, yet we do not affirm that the thing is *impossible.* Mr. Mayo was, at one of our former visits, about to operate on three or four patients; but at that time he had not faith enough to allow the Baron to lay his patients in ecstasy by his blue flame ere he flourished his knife.

Mr. Mayo seems to anticipate the smile of disbelief in these divining rods. They who have once smiled themselves, either in pity or in wonder, are highly prone to expect the smiles of others when they are converted. These questions are not essentially *psychological*, however they may fairly engage the time of the experimental philosopher; we will, therefore, pass on to the next subject, which is somewhat closer in its affinity to the manifestations of mind—Vampyrism! Of course the readers of Mr. Mayo must have anticipated a lucubration on the zoology of the Arabian Nights; that they were about to glut their curiosity by the natural history of the ghoul or the afrit. For even the modern definition of the vampyr by Horst is, *mutato nomine*, the very prototype of the monsters of those wondrous legends of the Sultaness Scheherazade:—

"A vampyr is a dead body which continues to live in the grave, which it leaves, however, by night for the purpose of sucking the blood of the living, whereby it is nourished and preserved in good condition, instead of becoming decomposed like other dead bodies."

And Fischer adds, the bite is speedily fatal, "although it leaves, in general, no mark upon the person. If the life of a victim be prolonged for a period, sooner or later he ends with becoming a vampyr himself—that is to say, he dies and is buried, but continues to lead a vampyr life in the grave, nourishing himself by infecting others, and promiscuously propagating vampyrism."

But with all this we may conclude that the longing for the supernatural will not be gratified, as Mr. Mayo sets himself very ingeniously to associate the demoniac fables of the vampyrs with two very interesting psychological questions, implicitly believing the *truths* of many an awful catastrophe, of which he selects two cases.

One story is that of a lover who, though deeply enamoured, blighted his passion because he believed himself doomed by the contamination of a vampyr. The lover dies; and in consequence of a prevailing illusion that he was a vampyr, and that the village was haunted by him, his body was disinterred forty days after his burial, and a stake being driven into his breast, "blood gushed forth, and the corpse uttered an audible groan." Subsequently, to clear the cemetery of the vampyrs, above a dozen bodies were thus exhumed, and although they had been under the sod for two or three weeks, many were as fresh and undecomposed as when they were buried, *fresh* blood being found in the chest. These appearances were amply sufficient, in ignorant or superstitious minds, to uphold, nay, to completely prove the truth of vampyrism; especially as one lady seemed to have become much fatter in her grave, in consequence of her nocturnal banquets of sanguisuction.

The other case is that of a man in the district of Kring, "who died, was buried, and became a vampyr, and, as such, was exhumed for the purpose of having a stake thrust through him. When they opened his grave, after he had been long buried, his face was found with a colour, and his features made natural movements, as if the dead man smiled. He even opened his mouth as if he would inhale fresh air. They held the crucifix before him, and called in a loud voice, "See, this is Jesus Christ who redeemed your soul from hell, and died for you." After the sound had acted on his organs of hearing, and he had connected, perhaps, some ideas with it, tears began to flow from the dead man's eyes. Finally, when after a short prayer for his poor soul, they proceeded to hack off his head, the corpse uttered a screech, and turned and rolled just as if it had been alive, and the grave was full of blood." For the solution of this wonder, Mr. Mayo leans altogether upon pathology, explaining the fact of the fresh and undecaying body in the grave by the occurrence of the death trance, citing the cases of the two colonels, Russell and Townsend, in analogous proof; and the withering influence of blind credence in the supernatural visitation, by allusions to the analogous occurrences of epidemic monomania. Of these awful instances of *life in death*, we have elsewhere fully dilated, discussing the phenomena of syncope, catalepsy, trance, &c., with the analogies of the hybernation of animals.

The horrors of premature sepulture, to which these unlucky vampyrs are doomed by mistake, is awful enough for the contemplation of the most prurient mind. To these mistakes Mr. Mayo offers these allusions:—

"A noise heard in a vault—the people, instead of breaking open the door, go for the keys, and for authority to act, and return too late: the unfortunate person is found dead, having previously gnawn her arm in agony. A lady is buried with a jewel of value on her finger: thieves open the vault to possess themselves of the treasure: the ring cannot be drawn from the finger, and the thieves proceed to cut the finger off; the lady, wakening from her trance, scares the thieves away and recovers. A young married lady dies, and is buried: a former admirer, to whom her parents had refused her hand, bribes the sexton to let him see once more the form he loved. The body opportunely comes to life at this moment, and flies from Paris with its first lover to England, where they are married."

The following cases more nearly concern the scalpel than the spade:—

"The Cardinal Espinosa, prime minister under Philip the Second of Spain, died, as it was supposed, after a short illness. His rank entitled him to be embalmed. Accordingly, the body was opened for that purpose. The lungs and heart had just been brought into view, when the latter was seen to beat. The cardinal, awakening at the fatal moment, had still strength enough left to seize with his hand the knife of the anatomist!"

The case of the Abbé Prevost, so often cited, is somewhat similar.

In a former number of this Journal, we have discussed the interesting point of the evidence of dissolution, &c. But we are scarce prepared, even now, to believe that out of 1200 (we quote from Frorieps Notizen) *presented* for burial at New York, six returned to life—one in every two hundred. This seems, however, very clear from the record of the mode of prevention, which consisted in the attachment of strings to the hands and feet of a corpse, kept eight days above ground in a coffin open at the head.

The inhuman mode of breaking the spell of vampyrism is truly demoniac, paralleled only by witch-burning and the tortures of the inquisition. The blind interpretation of the awful fact has been replete with unhappy inflictions. It has, as we have seen, caused the murder of its victim by impalement, and, by the mental contagion of superstitious terror, it has doomed a bevy of miserable beings to a half-living grave.

The first result of this blind credulity is spectral illusion, which Mr. Mayo calls "Unreal ghosts," citing the hacknied case of Nicolai and the vision of the Fetch in illustration. There are, however, much more interesting and impressive cases on record. The author travels here not a little out of the record. These cases of trance are the mere result of imitative monomania from intense mental impression—one only of the causes of spectral illusion. How rapidly even the *eyes* of a crowd may be made the fools of intelligence has been illustrated by many amusing anecdotes, but the *mind's* eye will also very soon run wild under the potent influence of a morbid sympathy. Among nuns, schoolgirls, and other supersensitive beings, the dance of the middle ages displayed the mania by wholesale.

In this *sympathy* lies much of the essence of magnetism, whether it be sympathetic *action* or sympathetic *thought*, which *is* the action of the brain. It requires no animal electricity to induce this, as if one body were positively, another negatively, electrified. The sensitive mind has its thought directed or impressed by, and brooding over, a subject; and this impression becoming general, the influence is irresistible. The simplest mode of analysis is to begin with the intense impression of *one* mind, by the concentration of thought or association. Such is the induction of phantoms, even in strong but imaginative minds; such the climax of his theological studies on Swedenborg; such the effect of the illuminated missals on Joan of Arc: a train of illusions fashioned according to the bent of disposition or *taste*—it may be according to the *moulding* of the organ of the mind.

On a weak or congested brain the effect would be one or other form of mania. These illusions we might aptly term religious cheromania, in which there is a microcosm of bright phantoms floating around the visionary, who is, we shall usually find, *quoad* the *action* of the illusive drama, pars magna. We might quote many instances displaying madness equal to that of Swedenborg and Theresa; but we will merely transcribe that of a youth of Königsberg, the natural son of a priest, from Mr. Mayo's book:—

He "had the impression that he was met near a crucifix on the wayside by seven angels, who revealed to him that he was to represent God the Father on earth, to drive all evil out of the world. The poor fellow, after pondering a long time upon this illusion, issued a circular, beginning thus—'We, John Albrecht Adelgreït Syndos, Amata, Kanemata, Kilhis Mataldis Schonal-Kilimundis Sabraudis Elioris Hyperarch, high-priest and emperor, prince of peace of the whole world, Hyperarch, king of the holy kingdom of heaven, judge of the living and the dead, God and Father, in whose divinity Christ will come on the last day to judge the world, Lord of all lords, King of all kings, &c."

This cheromaniac was thrown into prison and put to the torture; he had his tongue torn out with red-hot pincers, was cut in four quarters, and then burned under the gallows.

In the same category with these celestial maniacs, are others whose bright visions turn on the subject of their study or profession: such was Benvenuto Cellini, whose brows, he believed, were surrounded by halos of light, bright stars, &c., &c.; and, in the "Philosophy of Mystery," is recorded the case of a fine young officer, who, in enumerating his titles, exhausted the whole catalogue of dignities, royal, diplomatic, and military, and framed a list far more dignified than that of poor Albrecht, already quoted. These cases are evidence of the influence of the mind's constitution in the *fashioning* of illusions. On that mind that is tortured with remorse, or has been guilty of some dark deed, the visions will be of daggers, poison, or blood, as those of Macbeth and his almost supernatural wife.

Now these ghosts are all very intelligible, but they are accomplices *after* the fact, and therefore are indeed very useful in the matter of reform; ghosts act a very important part in the economy of morals. The ghost of the dream, for instance, will again and again glide into the chamber by night, and by patient perseverance will at length effect its object, even in the most obdurate hearts, just as continual dropping will wear away a stone.

Even the *prophetic* dream, as it is called—the death-fetch, although a morbid action, is not without its benefit. Many a wilful heart has been changed by this spectre of the wraith. "A young gentleman," we quote from Mr. Mayo, "told me that he was one evening at a supper party in college, when they were joined by a common friend on his return from hunting. They expected him, but were struck by his appearance: he was pale and agitated. On questioning him, they learned the cause. During the latter part of his ride home, he had been accompanied by a horseman who kept exact pace with him, the rider and horse being close fac-similes of himself and the steed he rode, even to the copy of a new-fangled bit, which he sported that day for the first time. He had in fact seen his double or fetch. His friends advised him to consult the college tutor, who failed not to give him some good advice, and hoped the warning would not be thrown away." It is added that, "it had made the ghost-seer, for the time at least, a wiser and a better man."

It is interesting to note how, from this disordered function, the same condition or posture will induce a repetition of the same train of thought. The dream or spectre, forgotten in the day time, will come again at night, like Hamlet's father, almost at the same hour. Of course, in the dark ages, this punctuality of visitation was referred to some spiritual law in the code of the government of shadows. WE know that the recumbent posture and its consequence, cerebral plethora, are the essence of all this.

In reference to the psychical illusions in Shakspeare's dramas, Mr. Mayo has a sort of random shot at the psychology of his genius. "While his great contemporary, Bacon, employed the lamp of his imagination to illuminate the paths to the discovery of truth, Shakspeare would, with random intuition, seize on the undiscovered truths themselves, and use them to vivify the conceptions of his fancy." May we presume to qualify somewhat the notion of this sentence, which will not obscure the millionth of a ray of Shakspeare's glory. Memory, in the minds of genius, will often stand them in stead of imagination. So was it with Walter Scott—the candour of his latter annotations having certainly plucked a plume or two from the wing of his ever-active fancy. So was it with Shakspeare, many of whose dramas were but different readings or splendid embellishments of real history. That Shakspeare was well read in Italian literature, Romeo or Shylock will prove; his English dramas show us that he had dipped deeply into British history, and his anachronisms and his eulogies show us how prone he was to warp its legends to his purpose. His classic reading, also, would have put him in possession of medical opinions, without our calling for the possession of clairvoyance to discover the mysteries of physiology; and the fact is, that Shakspeare's physiology is often defective, as in this passage:—

"And make each petty artery in this body
As hardy as the Nemæan lion's nerve,"

and some others.

Imagination! alas for the realm of Poesy, the region of bright shadows is shorn of half its sublimity in this matter of fact age. Fact is fast exterminating fiction from its romantic domain; for we now come to a very wondrous illustration of what we had always deemed a mere vagary of a poet's "midsummer madness." "There was a cottage in a village I could name, to which a bad report attached. More than one who had slept in it had seen at midnight the radiant apparition of a little child standing on the hearth-stone. At length suspicion was awakened. The hearth-stone was raised, and there were found beneath it the remains of an infant. A story was now divulged, how the last tenant and a female of the village had abruptly quitted the neighbourhood. The ghost was *real* and significant enough."

An old man disappeared near Cupar. A clergyman, twelve days after, looking over a wall, saw a bright and strange light. Here they dug, and found a buried body. It was identified, and the apprentice of the murdered man was tried and condemned. He confessed his crime and was executed.

Now, we confess we had believed these wondrous stories to be the result of rare coincidence or collusion; but no, the legends of spectral lights, and wraiths, and fetches, and churchyard ghosts, are no longer a mystery—no longer an illusion—no longer supernatural.

The haunted house of Athenodorus, in Pliny; the cauld lad of Hilton; the legends told by Baron Geramb and Lady Fanshawe, and a host of others, scattered throughout the pages of psychology, are now no fables.

But we have been anticipated in our *rationale* of these material phantoms. In 1794, they knew something about it, as we glean from a philosophical essay of that year: "The apparitions of souls departed do, by the virtue of their formative plastic power, frame unto themselves the vehicles in which they appear, out of the moisture of their bodies. So ghosts do often appear in churchyards, and that but for a short time—to wit, before the moisture is wholly dried up."

> "Such are those thick and gloomy shadows damp,
> Oft seen in charnel vaults and sepulchres,
> Lingering and sitting by a *new-made* grave."

But they did not dip deep enough into the mystery. It was reserved for Von Reichenbach to broach the true *philosophy* of the appearances. After quoting the record of Pfeffel, regarding the wondrous faculty of Billing, which has been often quoted, Mr. Mayo concludes his third chapter thus:—

"The explanation of this mysterious phenomenon has been but recently arrived at. The discoveries of Von Reichenbach announce the principle on which it depends. Among these discoveries is the fact that the O. D. force makes itself visible as a dim light, or waving flame, to highly sensitive subjects; such persons, in the dark, see flames issuing from the poles of magnets and crystals. Von Reichenbach eventually discovered that the O. D. force is distributed universally, although in varying quantities. But, among the causes which excite its evolution, one of the most active is chemical decomposition. Then, happening to remember Pfeffel's ghost story, it occurred to Von Reichenbach that what Billing had seen was possibly O. D. light. To test the soundness of this conjecture, Miss Reichel, a very sensitive subject, was taken at night to an extensive burying-ground near Vienna, where interments take place daily, and there are many thousand graves. The result did not disappoint Von Reichenbach's expectations. Whithersoever Miss Reichel turned her eyes, she saw masses of flame. This appearance manifested itself most about recent graves. About very old ones it was not visible. She described the appearance as resembling less bright flame than fiery vapour—something between fog and flame. In several instances the light extended four feet in height above the ground. When Miss Reichel placed her hand on it, it seemed to her involved in a cloud of fire. When she stood in it, it came up to her throat. The mystery has thus been entirely solved. For it is evident that the spectral character of the luminous apparition had been supplied by the seers themselves. So the superstition has vanished, but as usual, it veiled a truth."

Thus by these solutions are we psychologists cheated of half our vocation. We must not even contend with these materialists that the mind may possibly convert a flickering flame into a form, or turn to shape a wreath of smoke or vapour. *They* only, it seems, labour under a negative illusion, who do *not* possess the O. D. force.

Now might we suggest to the Sanitary Commission to inquire if the sense of smell is influenced by the O. D., and how far the O. Dorous effluvia darting to the brain in the localities of intra-mural sepulture, may not at this hour be rife with pestilence, especially to the sensitive possessor of this potent faculty.

The chapter on "real ghosts" leads us at once into the depths of deuteroscopia:—

"A Scottish gentleman and his wife were travelling four or five years ago in Switzerland. There travelled with them a third party, an intimate friend, a lady who sometime before had been the object of a deep attachment on the part of a foreigner, a Frenchman. Well, she would have nothing to say to him on the topic uppermost in his mind; but she gave him a good deal of serious advice, which she probably thought he wanted, and she ultimately promoted, or was a cognizant party to, his union with a lady whom she likewise knew. The so-married couple were now in America, and the lady occasionally heard from them, and had every reason to believe that both were in perfect health. One morning, on their meeting at breakfast, she told her companions that she had had a very impressive dream the night before, which had recurred twice. The scene was a room in which lay a coffin: near to it stood her ex-lover in a luminous, transfixed, resplendent state: his wife was by, looking much as usual. The dream had caused the lady some misgivings, but her companions exhorted her to view it as a trick of her fancy, and she was half persuaded so to do. The dream, however, was right notwithstanding. In process of time letters arrived, announcing the death, after a short illness, of the French gentleman, within the twenty-four hours in which the vision appeared."

"A late General Wynyard, and the late General Sir John Sherbroke, when young men, were serving in Canada. One day—it was daylight—Mr. Wynyard and Mr. Sherbroke both saw pass through the room where they sat a figure, which Mr. Wynyard then recognised as a brother far away. One of the two walked to the door, and looked out upon the landing-place, but the stranger was not there, and the servant, who was on the stairs, had seen nobody pass out. In time, news arrived that Mr. Wynyard's brother had died about the time of the visit of the apparition."

In a Durham paper there was an account of the disappearance of Mr. Smith, gardener to Sir Clifford Constable, who it is supposed had fallen into the river Tees, his hat and stick having been found near the water-side. The river had been dragged every day, but every effort so made to find the body proved ineffectual. On the night of Thursday, however, a person named Awde, residing at Little Newsham, a small village four miles from Wycliffe, dreamt that Smith was laid under the ledge of a certain rock below Whorlton Bridge, and that his right arm was broken. Awde got up early on Friday, and his dream had such an effect on him, that he determined to go and search the river. He rowed to the spot he had seen in his dream, and there, strange to say, with the very first trial that he made with his boat-hook, he pulled up the body of the unfortunate man with his right arm actually broken.

But we might cite cases multiform, more seemingly mysterious than these; and we have been wont to *explain* these visionary *truths* —i. e., the fulfilment of prophetic fantaisie, simply on the principle of coincidence, drawing, of course, a wide distinction between them and the phantom of guilty or innocent *memory*, in which we constantly perceive a complete concatenation of cause and effect.

We are informed of a ghost society at Cambridge, which collected above twelve well-authenticated instances of these comings to pass, or foreshadowings, or whatever they may be termed; but we hope this spectral league did not content itself with mere collection of these truths, (which they really are,) instead of analyzing and reasoning on the facts by the rule of chances.

Mr. Mayo rests confidently on the psychological elucidation of Zschokke, the late historian and novelist, that the selbstschau, or seer gift, is possessed by real beings, as well as by Allan Macaulay, Allan Bane, and Bryan. If such a faculty were possessed by many, the world would be a very awful field to act in. The deuteroscopia of the seer would be far more overwhelming than the inquisitorial callipers of the phrenologist. Who would willingly, or without dread, stand up before such a conjuror, who can thus see through you, in the literal sense of the term, expose the workings of your heart, and tell the very secrets of your soul? Yet such an inspired wizard was Zschokke.

"It has happened to me," he writes, "occasionally at the first meeting with a total stranger, when I have been listening in silence to his conversation, that his past life, up to the present moment, with many minute circumstances belonging to one or other particular scene in it, has come across me like a dream, but distinctly, entirely involuntary, and unsought, occupying in duration a few minutes. During this period, I am usually so plunged into the representation of the stranger's life, that at last I neither continue to see distinctly his face on which I was idly speculating, nor to hear intelligently his voice, which at first I was using as a commentary to the text of his physiognomy."

This Zschokke tells us that "on every occasion the confirmation of his 'dream vision' follows not without amazement on the part of those who gave it." "In a gamesome mood," he told his family the secret history of a sempstress, who had just left them, but whom he had never seen o known before. The hearers were astonished as well as himself at the exactness of his narration. On another occasion he narrated the private life of a young sceptic, who had ridiculed his power; among other things, "his school-years, his peccadilloes, and, finally, a little act of roguery committed by him on the strong box of his employer."

"I described the uninhabited room with its white walls, where to the right of the brown door there had stood on the table the small black moneychest. A dead silence reigned in the company during this recital, interrupted only when I occasionally asked if I spoke the truth. The man, much struck, admitted the correctness of each circumstance."

Mr. Mayo's credulity comes now more prominently into play.

"I shall assume it to be proved by the above crucial instance that the mind or soul of one human being can be brought in the natural course of things, and under physiological laws, hereafter to be determined, into immediate relation with the mind of another living person. *If* this principle be admitted"—(much virtue in this little *if*)—"it is adequate to explain all the puzzling phenomena of real ghosts and of true dreams," &c. &c.

So our learned author really believes in the principle of psychological projectiles, the mutual visitation of souls. But he does anticipate some scepticism notwithstanding.

"The school of psychological materialists will, of course, be opposed to this. They hold that the mind is but a function or product of the brain, and cannot therefore admit consistently its separate action. . . . If mind be the product of brain, it must be the conversion of so much brain weight into thought and feeling.

Psychological writers have endeavoured to reconcile this discrepancy. There is no paradox of belief when we gainsay the opinion that the mind cannot have, during the life of the body, even a momentary existence, independent of matter, and yet admit as a truth, that when this matter is in a state of repose, mind is perfectly passive *to our cognizance*.

There is no sophistry in affirming that soul and mind are the same under different combinations—the one not combined with or emancipated from matter, the other evinced through the medium of the brain. Thus we may have in the same essence diseased mind—i. e., brain, and pure immortal spirit.

The remaining subjects in the work are pathological and psychological TRUTHS, the legends which have raised them into mysteries being but exaggerations of fact.

The subject of trance is one of deep interest in psychology, the evidence of union between mind and matter being withdrawn by the sleep of the brain, not by the suspension of its *secretory* function. It assumes one of two states, its flights either far exceeding those of the waking moments, or being entirely suppressed.

With many of Mr. Mayo's reasonings on the mind's nature we fully agree; they tally with our own. Some of his preliminary conceptions, too, are put fairly enough. In his eight propositions consist much of the principle of psychology. In the fourth, however, the phenomena of clairvoyance, second sight, double consciousness, and transference of senses, are mingled in no little confusion. The psychological conditions of trance and somnambulism not rarely come under our notice. The normal manifestations of mind are altered in all.

We can readily believe that oppression, congestion, or concussion, will exhaust irritability by paralyzing the mind's organ, and then we observe its indications no longer. Undue excitement, on the contrary, may so exalt or concentrate the sense that its energies may become *hyper-acute*—to the unlearned intellect, preternatural. Even idiocy may thus display, on one concentrated point, a sign of something like supernatural intelligence.

We are inclined, generally, to grant the author his tether, but the principle of transference of senses is a parodox we cannot fathom. Who can believe that the beautiful diaphanous humours of the eye would have been formed if the faculty of vision, could in a moment be delegated to the scrobiculus cordis. And yet how else can we interpret this, the fourth proposition? "The abnormal relation (of mind and body) is conceivable—that is to say, a state in which a part or the whole of the mental faculties may occupy unaccustomed organs, or a part even be set entirely free." And again: "In almost all its forms it is easy to show that some of the mental functions are no longer located in their pristine organs." "The most ordinary change is the departure of common sensation from the organs of touch." So that there is no burlesque in Butler's lines—

"To chop and change intelligences,
So Rosicrucian virtuosis
Can see with ears, and hear with noses."

We are now arrived at the subject of mesmerism, and Mr. Mayo, in allusion to its double consciousness and clairvoyance, congratulates the profession on the acquisition of "a new remedy of gigantic power." We do not suppose that the author believes implicitly in the faculty of accurate diagnosis possessed by the ecstatic cataleptic; it is too ludicrous to write about. But that during and after the trance sleep, there is a sort of metempsychosis or metamorphosis of mind, there is no question. The disposition, habits, temperaments, taste, are essentially and diametrically changed. In our own practice, we have known more than one cataleptic girl turn from religion to licentiousness. One, the daughter of a milk-woman, was especially devoted to theological tracts and serious occupation; but, after the third attack of trance, her chief delight consisted in a visit to the gallery of the Surrey Theatre, among a very degraded class of beings. This monomania ended in suicide. That these symptoms depend on a change of action or circulation in the brain is very clear, and if these changes are effected so prominently in *natural*, spontaneous, or idiopathic catalepsy, we are prepared for a whole train of eccentric or remedial influences when such condition of brain is effected by artificial means. And it is as easy to believe the phenomena of double consciousness, if there be remissions of the *early* cataleptic symptoms, as to acknowledge that the waking and the dreaming mind will, in a few seconds, be influenced by thoughts and feelings of a totally opposite nature. Of this double consciousness, we will quote one interesting case of Dr. Barlow from Mr. Mayo's book, although we might cite cases of a like kind within our own knowledge.

" This young lady has two states of existence. During the time that the fit is on her, which varies from a few hours to three days, she is occasionally merry and in spirits, occasionally she appears in pain, and rolls about in uneasiness; but in general she seems so much herself that a stranger would not remark anything extraordinary. The fit leaves her suddenly, and she then forgets everything that has passed during it, and imagines that she has been asleep, and sometimes that she has dreamed of any circumstance that has made a vivid impression upon her. During one of these fits, she was reading Miss Edgeworth's tales, when she went for a few minutes to the window, and suddenly exclaimed, 'Mamma, I am quite well, *my headache is gone!*' Returning to the table, she took up the open volume, and said, 'what book is this?" seven or eight hours afterwards, when the fit returned, she asked for the book, went on at the very paragraph where she had left off, and remembered every circumstance of the narrative. She seems *conscious* of her state, for she said one day, 'Mamma, this is a novel, but I may safely read it; it will not hurt my morals, for when I am well I shall not remember a word of it.' "

And this is the summing up of Mr. Mayo on this young lady's case:—

" 1. The organs of sensation are deserted by their natural sensibility; the patient neither feels with the skin, nor sees with the eyes, nor hears with the ears nor tastes with the mouth.

"2. All these senses, however, are not lost; light and hearing, if not smell and taste, re-appear in some other part—at the pit of the stomach for instance, or the tips of the fingers.

"3. The patient manifests new perceptive powers. She discerns objects all round her, and through any obstructions, partitions, walls, or houses, and at an indefinite distance." (She beats the automaton chess-player hollow.) "She sees her own inside, as it were, illuminated, and can tell what is wrong in the health of others. She reads the thoughts of others, whether present or at indefinite distances. The ordinary obstacles of space and matter vanish to her. So likewise that of time; she foresees future events."

Now, if these propositions be true, it is clear that the whole system of physiology must be forthwith re-modelled. Perfect health—i. e., what we term "*Mens sana in corpore sano*,"—is a dream, and trance only exhibits the evidences of perfect wisdom; nay, it is little short of enchantment. There is an end to conjecture, and the cataleptic girl will enrobe herself in the academic toga, and propound all the mysteries of our hitherto obscure profession. She is the great mystery or medicine woman, and will reduce our pathology to a perfectly exact and logical science. She has nought to do but to "turn her visual power inwards," we quote the author's words, "and see her organs by the O. D. light they give out!"

The cases related by Dr. Petetin, of Lyons, (page 100,) are however, most interesting, and even if taken *cum granosalis* might tempt these inspired sybils instantly to found a college of witchcraft, for the purpose of ascertaining the measure of fidelity in lovers' hearts, with a thousand other inestimable discoveries. For this wondrous O. D. not only enables its possessor to use her knuckles as a lanthorn, or to see what she is writing with her elbow, but to predict battles and sieges, and to anticipate the thoughts and actions of mankind, their inspired minds being more lucid when the brain is converted into a sinecure, and the sense and senses are all transferred to the stomach! But these psychologists of the new birth are yet in a very splendid dilemma in the theory of fetch-ghosts, being uncertain which is the active, the ghost or the ghost-seer. Mr. Mayo, however, has his conjecture, at least, for he writes, "In the cases of ghosts and of dreams, coincident with the period of the death of an absent person, it seems simpler to suppose that the visit came from the other side; so the vampyr ghost was probably a visit made by the free part of the mind of the patient who lay buried in death-trance."

Mr. Mayo asks, "Are the sentiments and faculties suspended during sleep?" and answers "Certainly not!" Now we believe and have defined sleep to be essentially a suspension of the faculties. But in the *transition state* to and from sleep—the reverie of the soul, it might be termed—we have dreams passive and dreams active, or somnambulism. Some lapse into somnambulism at once during waking hours, and soldiers have been known in a long march to be unconscious of their walk. We should not term this *sleep*.

The most interesting story of *sleep-writing* is from the archbishop of Bordeaux, in the French Cyclopædia. We have known a few cases ourselves. Within a few weeks, we have been assured by a clergyman residing in a village near Oxford, that one of the best sermons he has written was composed in a night after he had arisen from sleep, during which he was perfectly unconscious of his task, and was bewildered in the morning when he saw it on his study table. Condorcet, Mackenzie, &c. &c., may term this reverie *sleep;* we do not.

We must wait, then, for more evidence of this O. D. force; but we may suggest that one of the effects most prolific of good would be its influence in dispelling those shadows of the mind which constitute religious delusion. We cannot resuscitate the victims of the inquisition; we cannot reinfuse the blood of England's martyrs; we cannot recal the persecutions and murders of the witches. If, however, there be any *use* in clairvoyance it would be to *clarify* the faculty of judgment, seeing that it displays thoughts and things in the very nakedness of truth. But the O. D. must surely cut both ways; it may obscure instead of enlightening, else whence the illusive visions of the enthusiast Swedenborg, Joan of Arc, Santa Theresa, Southcote, Thom, and the Mormonites, the socialists of the Agapemone, *et id genus omne ?*

Mr. Mayo puts forth these cases as trance, but the conditions of possession are often of a very *active* nature. Such were the ecstatic convulsions of the Cevennes, and those convulsionaires of 1727, in whom the clonic spasm was so extreme as to render the sensation of intensely hard blows agreeable! Such were also the lycanthropes or wehr-wolves of the 15th and 16th centuries, the jumpers, the jerkers, &c. &c. Analysis of these phenomena would indicate an intimate association of *imagination* and *self feeling;* the mad fancy has a conceit self-generated in the brain, or imparted by the contemplation of another, and this is combined with the self-feeling of the moment. Like the unconscious interval of a brace of analogous dreams, the fit being over, the illusion vanishes. Then comes another paroxysm: and lo, the mad thought *recurs,* the maniac is again a wolf, a demon, or a saint! All this is as physical as the delirium of fever.

We have not time nor space to analyse the nature of witchcraft—to point to the philters, the possets, the witchbroth, which the hags even of modern days employ for their demoniac purposes; nor to point to the secret nocturnal visitations to sensitive slumberers in nunneries and other large habitations, to which Mr. Mayo refers. Nor will we nauseate our readers by entering deeply into the *decies repetita* question of mesmerism. Let no one, however, smile in haughty scorn of this influence, because the power of transmission from one body to another has been so grossly exaggerated and misinterpreted, and displayed with such unblushing effrontery by the charlatan. We have ourselves, in a public arena, exposed the tricks of one of these impostors, who has since been convicted of sedition — the more harmless crime, perchance, of the two. There *is*, however, a truth in the physical effects of mesmerism, as clear as noon-day, unwillingly admitted, or totally denied by many, because its essence cannot be displayed. The *source* of its influence we believe to be purely psychical, as was that of Hohenlohe's miracles, Sir Kenelm Digby's sympathetic powder, &c. &c. We will not follow Mr. Mayo through his remarks on the analogies of æther and of chloroform, but if the narcotism of æther inhalation had been effected without a *tangible* therapeutic agent, what degree of credence would it have attained in the minds of those who had not *seen* its intense and speedy influence. The sceptic would not pause to inquire into the effect of moral or mental causes on the nervous system, and he would flout the records of its power as an imposition. The humility of real philosophy is more open to the truths of nature. He who feels that every natural action is a mystery, will be prepared to accept, at least to analyse and study, even that which may at the time be inexplicable. Such was the faith of the Hon. Robert Boyle, the advocate of Valentine Greatrex, yet he was laughed at for his credulity. No one, however, will now, with the incidents of the last ten years before him, argue that there was no truth in the agency of Greatrex, however his avarice or cunning may have exaggerated its power and extent. There is much, very much, to be yet studied, ere we unravel the mysteries of psychology, and no one can doubt its importance when he remembers that men have died under the impression that they were hung with a neckcloth loosely rolled round their neck, and that others have grown grey and decrepit in one night from intense impression on the mind!

The proselyte who proves too much does equal harm with the sceptic who admits nothing. The following aphoristic sentence would certainly scare a psychological student from Mr. Mayo's reading room:—"The O. D. force reaches us even from the stars, and the sun and the fixed stars are O. D. negative, and the planets and the moon O. D. positive." We see clearly, at least, that they have positively struck certain philosophers. If the lunatics go on in this course, we shall expect to see them not only casting nativities, but sending up kites to the moon, or even to the planet Mercury, our next neighbour, for a fresh supply of this astral element.

With all this freedom of comment, we will not deny that, call it what you will, there is a certain influence which induces effects beyond the pale of *every-day* experience, imparted to a predisposed system by some occult quality, or transmitted through the agency of another being; not merely by the paraphernalia of Mesmer, but by the simple hand-*waving* of Puységur and Victor. The state of sleep thus induced, may either prove a direct sedative remedy, tranquillizing some neuralgia already felt, or produce a state of unconsciousness to that pain which must otherwise have followed incision or other lesion of a nerve.

This is a brief statement of the *modus operandi* of Mesmerism:—

"The room should not be too light, very few persons should be present, the patient and the operator should be quiet, tranquil, composed, the patient should be fasting. The operator has then only to sit down before the patient, who is likewise sitting with his hands resting on his knees and gently closed, with the thumbs upwards. The operator then lays his hands, half open, upon the patient's, pressing the thumbs against those of the patient,—as it were taking thumbs; this is a more convenient attitude than taking hands in the ordinary way. The operator and patient have then only to sit still, an O. D. current is established, and if the patient is susceptible, he will soon become drowsy, and perhaps be entranced at the first sitting. Instead of this, the two hands of the operator may be held horizontally, with the fingers pointed to the patient's forehead, and either maintained in this position or brought downwards in frequent passes opposite to the patient's face, shoulders, arms, the points of the fingers being held as near the patient as possible, without touching."

Thus far, then, although we think the mental influence could be imparted with less form and ceremony, the thing seems rational and clear, and we can almost follow the account of Mr. Williamson's experiments with something like confiding interest. But we, by and by, read, "In ordinary trance the mind appears to gain new powers. The patient enters into a new relation with his Mesmeriser. He has no feeling, or taste, or smell of his own—feels, tastes, and smells everything that is made to tell on the senses of the operator." At this we feel somewhat puzzled, it goes further than our admission of the concentration of senses. Then comes this creative miracle of the operator—"He develops new organs of sensation;" then come the miracles of clairvoyance and eccentric mind, and among others, the absurd rhapsodies of Alexis. Now, we declare that those scraps of bombast go far to weaken our faith. We have heard a maniac come out with far more splendid moonshine; and as to the *new powers* of Alexis, there was so much guessing, and manœuvring, and failure, as to impress us at once with the shallowness of his pretensions.

We have thus fairly written our comments on the *truths* of psychological eccentricity, and, in candidly meeting the devotees halfway, we may hope to have convinced them of our willingness to look fairly and diligently into nature. But we are not yet, like Mr. Mayo—innoculated; nor have we imbibed more than a very mild dose of the Mesmeric malaria. Still the 'Letters' are not without interest, as the author often argues his points psychologically, however he may sometimes seem to lean directly away from admitted truths.

For ourselves, we believe that, as the alchymic crucible has unfolded many valuable treasures without realizing the golden dream, so Mesmerism, although failing as a science, may yet develop many psychological principles; but when we are told of its potency in diagnosis and remedy, of its solution of the most intricate mathe matical problems, of its foreshadowing of the fate of mankind, and, above all, of its inculcation of morality and virtue from the fear of having our inmost thoughts displayed, by the gifted creatures, naked to the world,—why, then, we close the book in pity at this humiliation of man's sovereign reason.

